# Extensive prevalence and significant genetic differentiation of *Blastocystis* in high- and low-altitude populations of wild rhesus macaques in China

**DOI:** 10.1186/s13071-023-05691-7

**Published:** 2023-03-17

**Authors:** Mengshi Yu, Yongfang Yao, Hongtao Xiao, Meng Xie, Ying Xiong, Shengzhi Yang, Qingyong Ni, Mingwang Zhang, Huailiang Xu

**Affiliations:** 1 College of Life Science, Sichuan Agricultural University, Ya’an, 625014 China; 2 College of Animal Science and Technology, Sichuan Agricultural University, Chengdu, 611130 China

**Keywords:** Rhesus macaque, *Blastocystis*, High altitude, Genetic diversity, Subtype

## Abstract

**Background:**

*Blastocystis* is a common intestinal protist with a wide range of hosts. Thus far, 38 subtypes have been identified. In recent years, wild animals have been confronted with habitat fragmentation as well as an increasing risk of zoonotic disease transmission due to human disturbance. Only limited data are available on *Blastocystis* infection and subtype distribution in wild rhesus macaques in China. The aim of the present study was to investigate the prevalence and genetic diversity of *Blastocystis* in nine wild rhesus macaque populations in China.

**Methods:**

A total of 276 faecal samples were collected from five high-altitude populations (high-altitude group [HAG]; 2800–4100 m a.s.l.) and four low-altitude populations (low-altitude group [LAG]; 5–1000 m a.s.l) of rhesus macaques. PCR-based analysis, using a new primer pair for the amplification of a 1690-bp sequence of the small subunit ribosomal RNA (SSU rRNA) gene, was used for prevalence and genetic diversity analysis.

**Result:**

Analysis of faecal samples revealed that *Blastocystis* infection was common in rhesus macaques, with an infection positivity rate of 80.1% (*n* = 221/276 samples). There was no significant difference (*P* = 0.121) in positivity rate between the LAG (84.3%) and HAG (76.8%). Overall, 33 haplotypes were obtained and classified into four subtypes (STs), of which three were potentially zoonotic subtypes (ST1, 29.7%; ST2, 16.7%; ST3, 31.9%) and one that was first identified in this study and named ST39 (12.0%). The STs were distributed differently among the rhesus macaque populations, except for ST3, which was found in all populations. Phylogenetic analyses revealed two major divergent clades of ST3 for the HAG and LAG. Genetic diversity analysis showed a high genetic diversity of ST3 (haplotype diversity: 0.846; nucleotide diversity: 0.014) in the rhesus macaques, but a high genetic differentiation (F_ST_ > 0.25) and a low gene flow (Nm = 0.09) between the HAG and LAG.

**Conclusion:**

Our study, which is the first investigation on *Blastocystis* infection in multiple wild rhesus macaque populations in China, indicates a potential risk of zoonotic transmission of *Blastocystis* in the study areas. *Blastocystis* ST3 showed high genetic diversity in wild rhesus macaques and significant genetic differentiation between the HAG and LAG. Our results provide fundamental information on the genetic diversity and prevalence of *Blastocystis* in wild rhesus macaque populations.

**Graphical Abstract:**

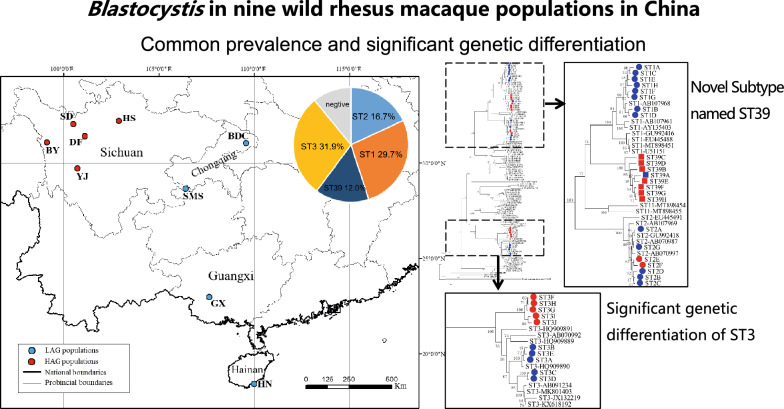

**Supplementary Information:**

The online version contains supplementary material available at 10.1186/s13071-023-05691-7.

## Background

*Blastocystis* is a genus of single-celled intestinal parasitic protists commonly found in humans [1] and detected in a wide range of animals, including non-human primates (NHPs), carnivores, rodents, birds and members of the mammalian orders Artiodactyla, Perissodactyla and Proboscidea [2]. The primary transmission route of *Blastocystis* is the faecal-oral route, and its prevalence in humans has been linked to hygiene status, exposure to animals and consumption of contaminated food or water [3, 4]. The pathogenic potential of *Blastocystis* remains controversial, with some studies reporting that infection with *Blastocystis* is associated with irritable bowel syndrome (IBS) [5], inflammatory bowel disease (IBD) [6] and cutaneous allergic disorders [7, 8]. However, recent studies on the microbiome have shown that *Blastocystis* may be a beneficial commensal rather than a pathogen [9, 10]. The commensalism or pathogenicity of *Blastocystis* could be related to subtype or even intra-subtype diversity [11].

Numerous molecular epidemiological studies have uncovered extensive genetic diversity within the genus *Blastocystis* [12]. To date, 38 subtypes (STs) of *Blastocystis* have been reported based on polymorphisms within the small subunit ribosomal RNA (SSU rRNA) gene [13–16]. However, based on current proposed guidelines, four subtypes (ST18–ST20 and ST22) are not recognised as valid subtypes [12]. In humans, 12 subtypes of *Blastocystis* have been reported (ST1-ST10, ST12 and ST14), with ST1–ST4 being the most prevalent subtypes [17, 18]. Analysing the genetic characteristics of subtypes and intra-subtype variants of *Blastocystis* in hosts will help to understand zoonotic transmission and the public health significance of this parasite. Molecular methods are more sensitive than traditional parasitological methods [19] and enable subtype identification based on PCR and sequencing.

*Blastocystis* does not seem to have a high host specificity, which provides appropriate conditions for zoonotic transmission [2]. In recent years, wild animals have been confronted with habitat fragmentation as well as an increasing risk of zoonotic diseases due to human disturbance, especially wild animals living in ecotourism areas. Ecotourism encourages close proximity that potentially increases zoonotic transmission [20]. This is more likely in the presence of a genetically similar host [21, 22]. The prevalence of *Blastocystis* in captive NHPs in China has been reported to range from 6.8% to 100%; these studies included rhesus monkeys in a zoo [23, 24] or laboratory [25, 26] but only one from a wild population [27]. However, it is necessary to investigate *Blastocystis* infection in wild NHP populations in China as parasite infection can be influenced by various complex factors. It has been reported that altitude and its related factors could affect the prevalence of parasites [28, 29], although the role of altitude in the prevalence of *Blastocystis* is unknown.

In China, wild rhesus macaque (*Macaca mulatta*) populations are widely distributed over a large range of altitudes, from sea level (Hainan Province) to 4000 m a.s.l. (Qinghai-Tibet Plateau [QTP] region), with large populations at altitudes in between (Sichuan, Yunnan, Guizhou and Guangxi provinces) [30]. The aim of this study was to investigate the prevalence of *Blastocystis* in nine wild rhesus macaque populations in China (Baiyu, Heishui, Seda, Yajiang, Daofu, Lingshui, Nanning, Fengjie, Jiangjin). Five of these populations live in ecotourism areas and have close contacts with humans because of being fed by tourists and staff at the tourism site. We recorded information on the altitude of each rhesus macaque population in detail and analysed the differences in prevalence of *Blastocystis* between high-altitude and low-altitude populations. Genetic diversity was explored based on PCR amplification and sequencing of the SSU rRNA gene.

## Methods

### Sample collection

**Fig. 1:**
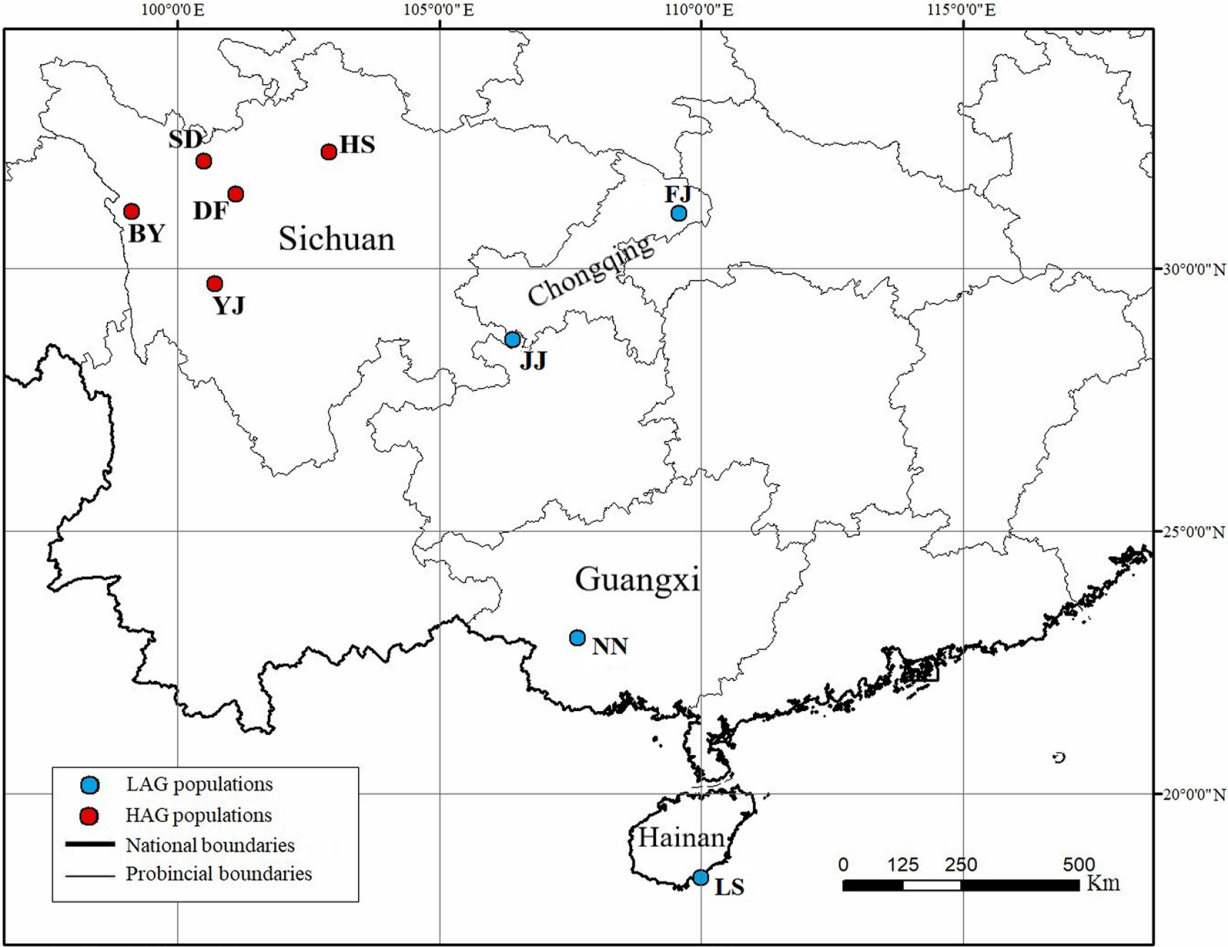
Specific locations at which samples were collected in this study. LAG, Low-altitude group; HAG, high-altitude group; SD, Seda; HS, Heishui; DF, Daofu; BY, Baiyu; YJ, Yajiang; JJ: Jiangjin; FJ: Fengjie; NN: Nanning; LS: Lingshui

To accurately evaluate the prevalence of *Blastocystis* in rhesus macaque, we collected 30–40 faecal samples from different individuals from each geographical population. The high-altitude group (HAG; > 2000 m a.s.l.) included five populations: Baiyu (BY; 4091 m a.s.l.;* n* = 36), Heishui (HS; 2846 m a.s.l.;* n* = 36), Seda (SD; 3500 m a.s.l.;* n* = 29), Yajiang (YJ; 3850 a.s.l.;* n* = 28) and Daofu (DF; 3200 a.s.l.;* n* = 26). The low-altitude group (LAG; < 1000 m) contained four populations: Lingshui (LS; 5 a.s.l.;* n* = 31), Fengjie (FJ; 158 m a.s.l.;* n* = 25), Jiangjin (JJ; 830 m a.s.l.;* n* = 29) and Nanning (NN; 158 m a.s.l.;* n* = 36) (Table 1). There were four geographic populations for which < 30 samples were collected due to the difficulty of sampling. In total, 276 faecal samples of rhesus macaques were collected from the nine populations from 2016 to 2020 (Fig. 1). The HAG populations were located in the QTP region in Sichuan Province, which is characterised by low temperature, low oxygen levels and high ultraviolet luminescence. The four LAG populations were located in Hainan Province, which has a low-altitude tropical monsoon climate, and in Chongqing and Guangxi provinces, which both have a subtropical monsoon climate. The rhesus macaques of the HS, HN, FJ, JJ and NN populations live in ecotourism areas with wild living status but have close contact with humans and are frequently fed by tourists or staff at the tourism site. The other four populations, BY, SD, YJ and DF, live close to or overlap with villages and pastoral areas. To largely avoid the repeated collection of samples from the same individual, we chose a continuous period of time in a day to follow the rhesus macaque population and sampled through direct observation. Difference samples collected at a distance > than 5 m from each other were recorded as samples from different individuals. Using sterile disposable gloves, Faecal samples were collected by hand, with the person wearing sterile disposable gloves, immediately after defecation, transported to the laboratory in an ice box and stored in a refrigerator at − 80 °C until DNA extraction.

**Table 1:** Geographic information and altitude of rhesus macaque populations

Province	Location	Population identification code	Coordinates		Altitude (m a.s.l.)	No. of faecal specimens collected
			Latitude	Longitude		
Sichuan	Baiyu	BY	31°4′53″	99°7′15″	4091	36
	Heishui	HS^a^	32°12′53″	102°53′19″	2846	36
	Seda	SD	32°2′14″	100°30′22″	3500	29
	Yajiang	YJ	29°42′0″	100°43′12″	3850	28
	Daofu	DF	31°24′37″	101°6′42″	3200	26
Hainan	Lingshui	LS^a^	18°23′45″	109°58′52″	5	31
Guangxi	Nanning	NN^a^	22°57′32″	107°37′48″	158	36
Chongqing	Fengjie	FJ^a^	31°2′27″	109°34′39″	180	25
	Jiangjin	JJ^a^	28°38′41″	106°24′20″	830	29
Total						276

^a^Population living in ecotourism area

### DNA extraction

The genomic DNA of each faecal sample was isolated using the QIAamp Fast DNA Stool Mini Kit (Qiagen, Hilden, Germany), following the instructions of the manufacturer. The obtained DNA specimens were stored at − 20 °C until PCR analysis.

### PCR amplification

*Blastocystis* was identified by using nested PCR amplification. The primer pair RD3 (5’-GGG ATC CTG ATC CTT CCG CAG GTT CAC CTA C-3’) and RD5 (5’-GGA AGC TTA TCT GGT TGA TCC TGC CAG TA-3’) was used for the first PCR [12]. According to recently proposed guidelines [12], novel subtypes should be based on ≥ 80% of the approximately 1800-bp SSU rDNA gene. We designed a new primer pair based upon multiple sequence alignment of the SSU rDNA gene (BLA2F: 5’-CGG TAG TCA TAC GCT CGT CTC A-3’; BLA2R: 5’-ACC TAC GGA AAC CTT GTT ACG AC-3’) and used it for the secondary PCR, allowing the amplification of a 1690-bp fragment of the SSU rRNA gene. The cycling conditions were: an initial cycle at 94 °C for 3 min; followed by 35 cycles of 94 °C for 15 s, 65 °C (primary PCR) or 60 °C (secondary PCR) for 15 s and 72 °C for 15 s; followed by 1 cycle at 72 °C for 5 min. Each specimen was analysed twice using 2 µl of extracted DNA per PCR, and 2× PCR mixture (CoWin Biosciences Company, Cambridge, MA; USA) was used for all PCR amplifications. All secondary PCR products were examined by electrophoresis in 1.5% agarose gels containing ethidium bromide. The new primer pair was used for all DNA samples, and the sequences were used for phylogenetic and genetic diversity analyses.

### Sequencing and cloning

All positive PCR products were sequenced at Tsingke Biotech (Chengdu, China) by bidirectional sequencing. The raw sequences were edited manually using Lasergene Seqman software version 7 (https://www.dnastar.com/; DNASTAR, Madison, WI, USA). All sequences were compared with reference sequences available in GenBank, using the Basic Local Alignment Search Tool (BLAST). According to the recommendations for annotating new subtypes, *Blastocystis* subtypes were determined by exact match or an identity > 96% against reference subtypes, and all sequences obtained from this study were analysed by BLAST separately at each end [12].

Raw sequences showing stretches of indistinguishable peaks were considered to be mixed infections with different subtypes and were further cloned. Positive PCR products from the nested-PCR were extracted from the electrophoresis gel using the TIANgel Midi Purification Kit (Tiangen Biotech, Beijing, China). Then, 2 µl of DNA was used with the pClone007 vector (Tsingke Biotechnology, Beijing, China). Subsequently, between 5 and 10 colonies from each transformation were selected and sent to Tsingke Biotechnology for subculturing, plasmid purification and sequencing.

All 33 representative sequences generated in this study were submitted to GenBank under accession numbers OP563821–OP563853.

### Phylogenetic and genetic diversity analysis

The full-length *Blastocystis* reference sequences of known valid subtypes (ST1-ST17, ST21 and ST23-ST34) were downloaded from the reference database at http://entamoeba.lshtm.ac.uk/blastorefseqs.htm (updated 19 Nov 2021) and GenBank. Nucleotide sequences were aligned with the MUSCLE algorithm, phylogenetic analyses were performed using neighbour-joining (NJ) and maximum-likelihood (ML) methods based on 1690-bp sequences and genetic distances were calculated with the Kimura 2-parameter model using MEGA X, which was also used for evolutionary analysis to establish divergence between sequences (pairwise distance) (http://www.megasoftware.net/). Bootstrapping with 1000 replicates was used to determine support for the generated clades.

To investigate the genetic diversity of *Blastocystis* and the genetic differentiation between the LAG and HAG, DnaSPv4 [40] was used to conduct the genetic analysis of *Blastocystis* ST3, which was found in all populations. We included these indices: number of segregating sites (S), haplotype diversity (Hd), nucleotide diversity (π), average number of pairwise nucleotide differences (K), gene flow (Nm), genetic differentiation index (F_ST_), Fu's* Fs* and Tajima’s* D* test.

### Statistical analysis

Differences in the prevalence of *Blastocystis* among the HAG and LAG and among geographical populations were analysed by Chi-square test (*χ*^2^) in SPSS version 22.0 (SPSS IBM, IBM Corp., Armonk, NY), and differences at *P* < 0.05 were considered to be statistically significant.

## Results

### Prevalence of* Blastocystis* in rhesus macaque populations

A total of 276 faecal samples were collected from the nine rhesus macaque populations, and *Blastocystis* was detected in 221 samples (80.1%) (Table 2). *Blastocystis* prevalence varied significantly among the nine populations, being significantly lower in populations SD (55.2%), DF (42.3%) and LS (64.5%) (*P* < 0.001). The highest prevalence of *Blastocystis* was found in population NN (97.2%), followed by JJ (96.6%) and YJ (96.4%). The prevalence rate of *Blastocystis* did not significantly differ between the HAG (76.8%) and LAG (84.3%) (*P* = 0.121) (Additional file 1: Table S1).

**Table 2:** The prevalence rate and subtype distribution of *Blastocystis* in the rhesus macaque populations

Population	No. of faecal specimens collected	No. of *Blastocystis*-positive faecal specimens (%)	*P* value	No. of subtypes identified (%)				Mixed infections (*n*)
				ST1	ST2	ST3	ST39	
BY	36	33 (91.7)	0.303	–	–	24 (66.7)	11 (30.6)	ST39/ST3 (2)
HS^a^	36	32 (88.9)	0.164	–	24 (66.7)	7 (19.4)	1 (2.8)	–
SD	29	16 (55.2)	0.000	–	1 (3.4)	10 (34.5)	5 (17.2)	–
YJ	28	27 (96.4)	0.856	–	6 (21.4)	16 (57.1)	5 (17.9)	–
DF	26	11 (42.3)	0.000	–	2 (7.7)	3 (11.5)	8 (30.8)	ST39/S2 (1), S2/S3 (1)
LS^a^	31	20 (64.5)	0.000	12 (38.7)	6 (19.4)	4 (12.9)	–	S1/S2 (1), S1/S3 (1)
FJ^a^	25	19 (76.0)	0.011	12 (48.0)	–	15 (60.0)	–	S1/S3 (8)
JJ^a^	29	28 (96.6)	0.876	27 (93.1)	–	4 (13.8)	–	S1/S3 (3)
NN^a^	36	35 (97.2)	Reference	31 (86.1)	7 (19.4)	5 (13.9)	3 (8.3)	S1/S2 (4), S1/S3 (2), S2/S3 (1), S1/S2/S3 (1), S1/ST39 (2)
HAG (> 2000 m)	155	119 (76.8)	0.121	–	33 (21.3)	60 (38.7)	30 (19.4)	
LAG (< 2000 m)	121	102 (84.3)		82 (67.8)	13 (10.7)	28(23.1)	3 (2.5)	
Total	276	221 (80.1)		82 (29.7)	46 (16.7)	88 (31.9)	33 (12.0)	27 (9.8)

*ST* Subtype

^a^Population living in ecotourism area

### Distribution of and mixed infection with* Blastocystis* subtypes in rhesus macaque populations

Sequence alignment analysis of the SSU rRNA gene (approx. 1690 bp) confirmed 33 haplotypes from 221 *Blastocystis*-positive samples, and four subtypes were further identified, including the three potentially zoonotic subtypes ST1, ST2 and ST3 and one novel subtype. In accordance with the suggestion of Stensvold et al. and the members of the International *Blastocystis* Network [12]**,** the novel subtype was named ST39. The distribution and prevalence of these subtypes were different (Table 2): ST1 was only detected in LAG populations (67.8%); ST2 had the lowest prevalence (16.7%) of the known subtypes and was distributed in six populations; whereas ST3 was the most highly prevalent subtype (31.9%) and was distributed in all populations. In our study population, ST3 was the dominant subtype in HAG (38.7%). The novel subtype ST39 was identified in 33 samples (12.0%) from five HAG populations and one LAG population. There was a statistically significant difference in the prevalence of all subtypes (*P* < 0.05) between the HAG and LAG (Additional file 1: Table S1).

Mixed infection with different subtypes was found in 27 samples (27/318, 9.8%), mainly from LAG populations (23/27) (Table 2). Most samples found to have mixed infections had infection with two subtypes, with ST1 was the most common subtype (22/27), followed by ST3 (19/27). Only one sample from NN was had a mixed infection with three subtypes (ST1/ST2/ST3).

### Validation of the novel ST39 and variations of* Blastocystis* intra-subtype

Using the new primer pair, sequences of the SSU rRNA gene were successfully obtained for the known and novel subtypes. The availability of the new primer pair was verified: the 600-bp barcode regions of the sequences amplified by using the new primers were 100% matched with the fragment using the standard primers [12]. A total of 33 haplotypes were obtained from 352 sequences (Additional file 2: Table S2).

**Fig. 2:**
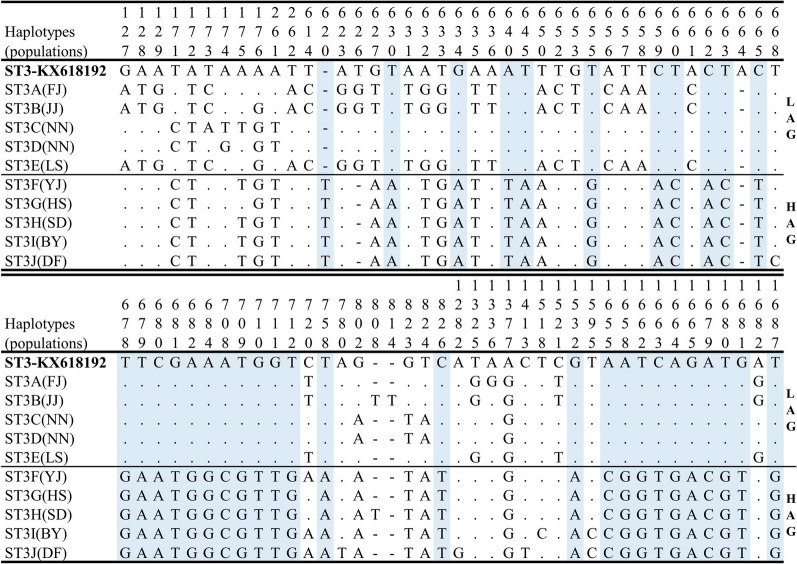
Sequence variation among ST3 sequences. The exclusive variable sites of HAG haplotypes are marked with blue shading. ST, Subtype

The novel subtype ST39 had eight haplotypes, ST39A–H. The most closely related reference sequence for ST39 was ST1-AB107968 (from *Cercopithecus aethiops* in Japan), with 95.3–95.4% nucleotide identity, which is in compliance with the proposed guidelines that new subtypes should normally differ by ≥ 4% from previously known subtypes [12]. The ST1 haplotypes (ST1A–H) showed 99.3%–99.6% identity with the reference sequence ST1-AB107968. The ST2 haplotypes (ST2A–G) showed 99.4%–99.8% identity with the reference sequence ST2-AB070987 (from human in Japan). The ST3 had 10 haplotypes, ST3A–E, which were obtained from the LAG, and ST3F–J were obtained from the HAG. Significantly, there was a large difference in sequence variation in ST3 haplotypes between the LAG and HAG (96.1%–97.4% nucleotide identity). The sequence ST3-KX618192 (from human in Singapore) was the closest reference sequence to haplotypes of the LAG, with 98.3%–99.5% nucleotide identity and 97.1%–97.5% identity with haplotypes of the HAG. Single nucleotide polymorphism (SNP) analysis detected 35 exclusive variable sites in ST3 haplotypes of the HAG (Fig. 2). The distribution of haplotypes showed that each subtype of most populations had one or two haplotypes and that only the NN population had four ST1 haplotypes and four ST2 haplotypes. The JJ and FJ populations shared the haplotype ST1C, whereas the YJ, HS and SD populations shared the haplotype ST2E (Additional file 1: Table S1).

### Phylogenetic analysis

**Fig. 3:**
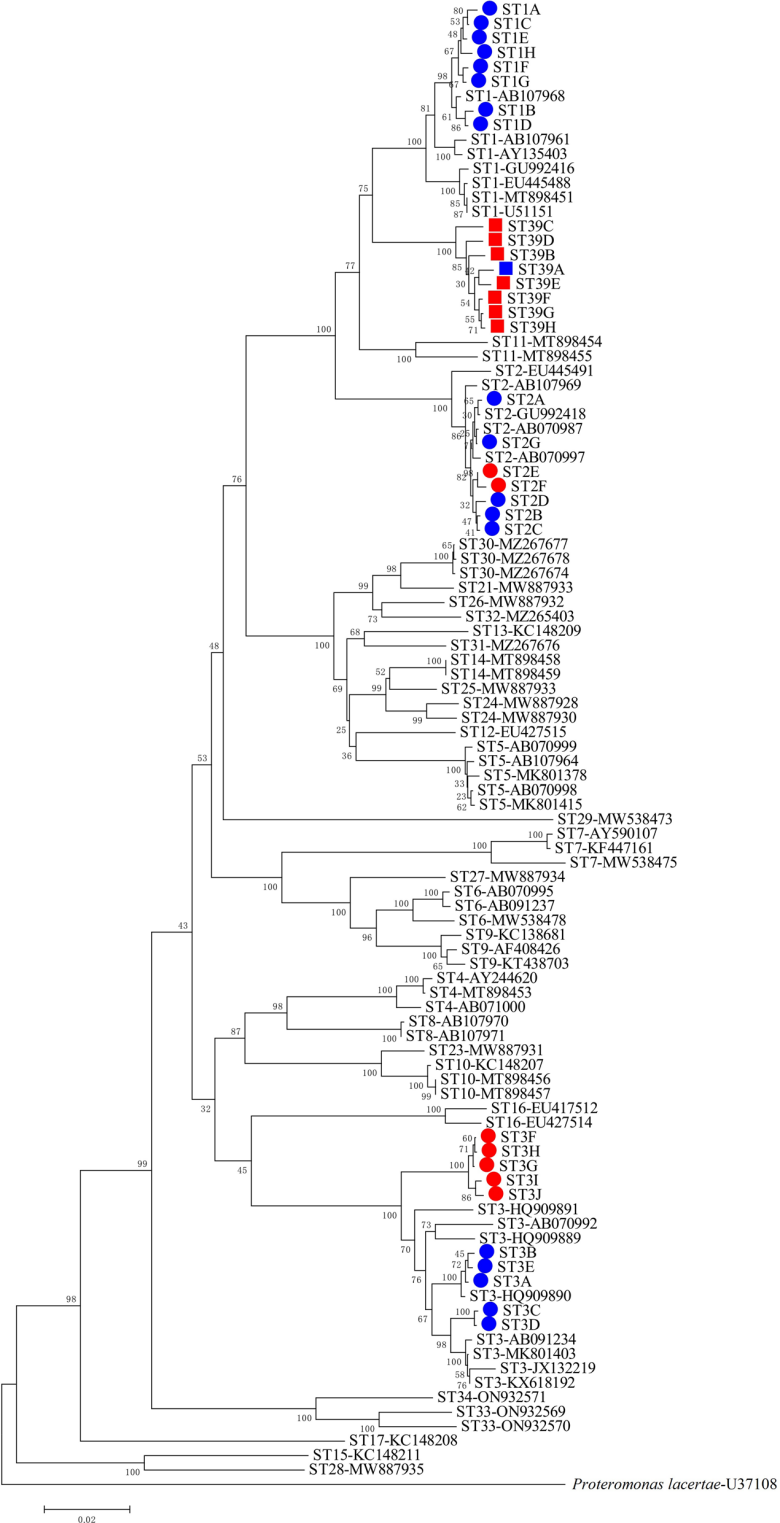
Molecular phylogenetic relationships among *Blastocystis* in the present study and representative reference sequences as inferred by the neighbour-joining tree based on the small subunit ribosomal RNA (SSU rRNA) gene (1690 bp). Genetic distances were calculated using the Kimura two-parameter model. The numbers on the branches are the percentage bootstrap values of 1000 replicates. Evolutionary analyses were conducted in MEGA X; *Proteromonas lacertae* was used as the outgroup taxon to root the tree. Novel subtypes are represented with a square and other subtypes with a circle; the sequences obtained from the LAG are shown in blue, and those from the HAG are shown in red

Phylogenetic analysis was performed using 33 representative sequences (approx. 1690 bp) and full-length reference sequences (ST1–ST17, ST21 and ST23–ST34), with *Proteromonas lacertae* included as an outgroup based on the NJ method (Fig. 3): the novel subtype ST39 clustered with ST1, ST2 and ST11. The bootstrap value was 75 for the clades formed by ST39 and ST1 and 77 for the clades formed by ST39/ST1 and ST11. The sequences obtained in this study clustered with currently recognised subtypes. Significantly, the ST3 haplotypes obtained from populations in the HAG (ST3F–ST3J) clustered in a sub-clade and were separated from another sub-clade which was formed by all reference sequences of ST3 and haplotypes of the LAG (ST3A–ST3D), with a bootstrap value of 100. Pairwise distance comparisons were used to evaluate the percentages of the shared sequence identity of ST39 with all reference sequences (Table 3): the percentages of the average sequence similarity between ST39 and the known subtypes ranged from 83% to 95%. In addition, the ML tree (Additional file 3: Figure S1) showed topologies similar to the NJ tree, providing further support for the validity of the novel subtype and the identification of the known subtypes.

**Table 3:**
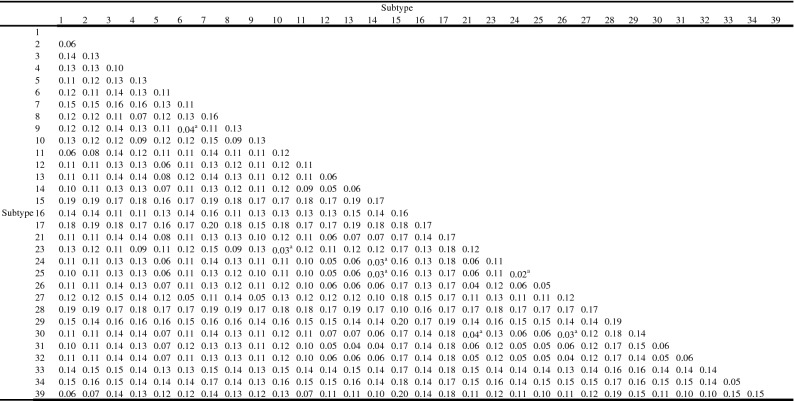
Pairwise distances between *Blastocystis* subtypes (1690bp SSU rRNA gene sequences) showing the average number of base substitutions per site. Analyses were conducted using the Kimura 2-parameter model and included 33 representative sequences and 66 reference sequences.

Analyses were conducted using the Kimura 2-parameter model and included 33 representative sequences and 66 reference sequences

^a^Pairwise distance ≤ 0.04

### Genetic diversity of* Blastocystis* ST3 in rhesus macaque populations

The results of the genetic analysis showed a high level of genetic diversity in all populations (Table 4), with 78 segregating sites (S), Hd of 0.846, π of 0.014 and a K of 23.823. The genetic diversity indices of ST3 in the LAG were higher than those in HAG, except for the close value of Hd. All Tajima’s* D* and Fu’s* Fs* values were positive, and the differences in the neutrality indices were not statistically significant. The F_ST_ and Nm values of ST3 between the LAG and HAG were 0.732 and 0.09, respectively, revealing a high degree of genetic differentiation and a low gene flow.

**Table 4:** Genetic diversity indices of *Blastocystis* ST3 in the HAG and LAG and in all populations

Populations	*N*	h	S	Hd ± SD	π ± SD	K	Tajima’s D	Fu’s* FS*	F_ST_	Nm
HAG	60	5	8	0.737 ± 0.032	0.001 ± 0.001	2.001	0.438 ns	2.935 ns	0.732	0.09
LAG	29	5	35	0.701 ± 0.054	0.008 ± 0.004	10.852	1.307 ns	14.361 ns	–	–
All populations	89	10	78	0.846 ± 0.017	0.014 ± 0.001	23.823	2.832 ns	35.127 ns	–	–

*N* number of sequences, *h* number of haplotypes, *S* number of segregating sites, *Hd* haplotype diversity,* SD* standard deviation, *π* nucleotide diversity, *K* average number of nucleotide differences,* F*_*ST*_ genetic differentiation index,* Nm* gene flow, *ns* not signifcant

## Discussion

*Blastocystis* is distributed worldwide as a common intestinal protozoan parasite that can infect a range of hosts; wild and domestic animals are potential reservoirs for human infection [2]. The prevalence of *Blastocystis* in wild NHPs in China has been poorly studied, only one study reporting the infection by *Blastocystis* ST5 in a golden snub-nosed monkey (*Rhinopithecus roxellanae*) in a natural reserve [27]. The present study is the first to investigate *Blastocystis* in multiple wild rhesus macaque populations in China. Our results provide data on the prevalence and distribution of *Blastocystis* subtypes, and data on the genetic diversity of *Blastocystis* ST3.

The findings of the present study indicate that *Blastocystis* is highly prevalent in wild rhesus macaques (80.1% of faecal samples; 221/276), with significant differences in prevalence among the nine wild rhesus macaque populations. A previous study reported that *Blastocystis* prevalence in captive macaques in China ranged from 5.6% to 96.6% [2, 24], but only a few studies have investigated *Blastocystis* in wild populations of macaques. In Thailand, the prevalence of *Blastocystis* in 14 wild *Macaca fascicularis* populations was 41.9% [31], which is lower than that found in the present study population. This variation in the prevalence of *Blastocystis* may be attributed to the difference in environmental and geographical factors [3]. Various factors influence the prevalence of parasites in wild animals. During sampling, we observed that rhesus macaques living in ecotourism areas were frequently fed by tourists and staff at the tourism site. Feeding is one of the most popular activities in ecotourism, providing tourists with an opportunity to come into contact with wild animals. A previous study reported that feeding sites in ecotourism areas might indirectly affect parasite transmission in Yunnan snub-nosed monkeys [32]. For wild rhesus macaques, close contact with humans and gathering at feeding sites may lead to zoonotic transmission and a high prevalence of parasites.

In our study, there was no significant difference in the prevalence of *Blastocystis* between the LAG (84.3%) and HAG (76.8%). Normally, the warm and humid climate of low-altitude areas may be suitable for parasite survival, leading to more chances for the parasite to colonise the host. The high prevalence of *Blastocystis* in the HAG indicates that at high-altitude areas, the parasite may be not largely impacted by environmental stress. At present, there are no relevant studies on the impacts of environmental factors on *Blastocystis* infection. Research estimating the effects of natural environmental factors, human interventions and socioeconomic conditions on parasitic diseases may inform disease prevention and control strategies [33]. In our future research, we will focus on additional factors influencing *Blastocystis* or other zoonotic parasite infections, with the aim to provide suggestions for protecting wild rhesus macaques from parasite infections.

In the present study, we identified three known subtypes, ST1–ST3, and one novel subtype, ST39, from wild rhesus macaques. Previous studies reported infections with ST1–ST5, ST7–ST11, ST13, ST15 and ST19 in NHPs, with ST2 being the most common subtype in wild NHPs, followed by ST1 [2]. However, we found that ST2 had the lowest prevalence of the known subtypes in rhesus macaques (Table 2). In addition, the prevalence of subtypes differed significantly (*P* < 0.05) between the HAG and LAG, most likely because of the different distributions of subtypes: ST3 was found in all populations, whereas ST1 was only present in the LAG. A previous study suggested that water and soil were the main reservoirs of *Blastocystis* in a community in Thailand and that *Blastocystis* might be transmitted by contaminated food and water [34]. Since our investigation is only based on faecal samples of rhesus macaques, it is impossible to prove zoonotic transmission and definitively identify the reservoirs of these subtypes in the study areas. However, some rhesus macaque populations live near villages or overlap with pastoral areas, thereby potentially using the same water and food sources as the villagers and free-range livestock. Although *Blastocystis* infection in humans or livestock was not investigated, considering that ST1–ST3 are common subtypes in human infection [15], the high prevalence of these subtypes in rhesus macaques may indicate a potential risk of zoonotic transmission. Further competitive interactions among *Blastocystis* subtypes may cause differences in the prevalence of subtypes. Such interactions can be mediated by factors such as the abundance of host resources, host immune responses and specific parasite strategies for survival, growth and reproduction [35]. However, to date, there has been no study on the competitive interactions of *Blastocystis* subtypes*.*

In the present study, 33 haplotypes were obtained from the four *Blastocystis* subtypes, indicating the remarkable genetic diversity of *Blastocystis* in rhesus macaque populations. In most populations, the number of haplotypes was limited. By comparison, NN had more haplotypes of ST1 and ST2, which may indicate multiple introduction events of *Blastocystis* by infected tourists. However, the sequences published in GenBank are not the same as those of the haplotypes obtained from rhesus macaques, and the existing data could not prove zoonotic transmission in rhesus macaques. Another speculation is that this could be the result of population founder effects. Regarding the finding of the same haplotypes in different populations, we speculate that the same haplotypes may have colonised rhesus macaques before the migration of the rhesus macaque population.

The results of the genetic analysis showed that *Blastocystis* ST3 has a high genetic diversity, with significant genetic differentiation between the LAG and HAG. This leads us to infer that isolation is caused by a large geographical distance. Results from previous studies suggest that the high molecular diversity of *Blastocystis* may be correlated with high host multiplicity and a coevolutionary history with the host [36, 37]. The significant genetic differentiation may indicate the long-term evolution and prevalence of ST3 in rhesus macaque populations or the areas of HAG, possibly explaining the large number of exclusive variable sites in haplotypes of the HAG. Based on the barcoding region of the SSU rRNA gene (approx. 600 bp), the significant negative Tajima’s* D* and Fu’s* Fs* values suggest a recent demographic expansion of ST3 in Asia [35]. However, data only from rhesus macaques and limited study areas may not reveal the demographic expansion of *Blastocystis*. Furthermore, genetic diversity and population diversification might play a key role in the generalist profiles and cross-species infections of some parasites [38, 39]. The use of more genetic markers will facilitate an understanding of the genetic diversity of *Blastocystis.* Recently, MinION long-read sequencing was used in the generation of accurate full-length sequences of the SSU rRNA gene [14]. Analysis of full-length sequences may result in the detection of more SNPs and haplotypes. High-throughput sequencing is more sensitive to the presence of mixed infections than Sanger sequencing and cloning [13]. The results regarding the mixed prevalence rate might be limited by the number of clones for sequencing in our study. Using the new-generation sequencing technology will facilitate genetic research on *Blastocystis.*

In this study, we identified and validated one novel subtype, which we named ST39. In addition, phylogenetic analysis based on the SSU rRNA gene indicated that ST39 and ST1 have a close genetic relationship. ST1 is a zoonotic subtype that is associated with asymptomatic infection [17]. At present, the host adaptation and pathogenicity of ST39 are unknown, and future studies should use both in vitro and in vivo experiments to investigate the pathogenicity of ST39. Furthermore, comparative genome sequence analysis, transcriptomics, immunological assays and microbial and metabolic profiling of specific hosts will greatly increase our knowledge of *Blastocystis* biology and its relevance to the host.

## Conclusions

This is the first study on *Blastocystis* infection and genetic diversity in multiple wild rhesus macaque populations in China. The studied populations were located in high- and low-altitude areas. Infections with the zoonotic subtypes ST1–ST3 are common in rhesus macaques, and we assume that there may be a potential risk of zoonotic transmission of *Blastocystis* in the study areas. We observed different distributions of *Blastocystis* subtypes and genetic differentiation of ST3 between LAG and HAG. The novel subtype ST39 was identified and validated, and its host adaptation and pathogenicity should be issues of concern. The present results provide fundamental information on genetic data and the prevalence of *Blastocystis* in wild rhesus macaque populations.

## Supplementary Information

**Additional file 1: Table. S1.** Chi-square test for the prevalence between HAG and LAG.

**Additional file 2: Table. S2.** Distribution, positive number and accession numbers of subtype haplotypes.

**Additional file 3: Figure S1**. Phylogenetic relationships among* Blastocystis* SSU rRNA gene sequences (1690 bp) in the present study (represented with a black circle) and representative reference sequences.* Proteromonas lacertae* was used as outgroup taxon to root the tree. Analysis was conducted by a maximum likelihood method. Genetic distances were calculated using the Kimura two-parameter model. **Additional file: 3 Fig. S1.** Phylogenetic relationships among *Blastocystis* SSU rRNA gene sequences (1690 bp) in the present study (represented with a black circle) and representative reference sequences. *Proteromonas lacertae* was used as outgroup taxon to root the tree. Analysis was conducted by a maximum likelihood method. Genetic distances were calculated using the Kimura two-parameter model.

## Data Availability

Data supporting the conclusions of this article are included within the article. Representative nucleotide sequences generated in this study were deposited in the GenBank database under the accession numbers OP563821–OP563853.

## Abbreviations

HAG: High-altitude group
IBD: Infammatory bowel disease
IBS: Irritable bowel syndrome
LAG: Low-altitude group
NHPs: Non-human primates
QTP: Qinghai-Tibet Plateau
SSU rRNA: Small subunit ribosomal RNA
ST: Subtype
