# Supplementary figures and images for: Extensive prevalence and significant genetic differentiation of Blastocystis in high- and low-altitude populations of wild rhesus macaques in China
Source: Parasit Vectors. 2023 Mar 17;16:107. doi: 10.1186/s13071-023-05691-7 (PMC10022093; doi:10.1186/s13071-023-05691-7)

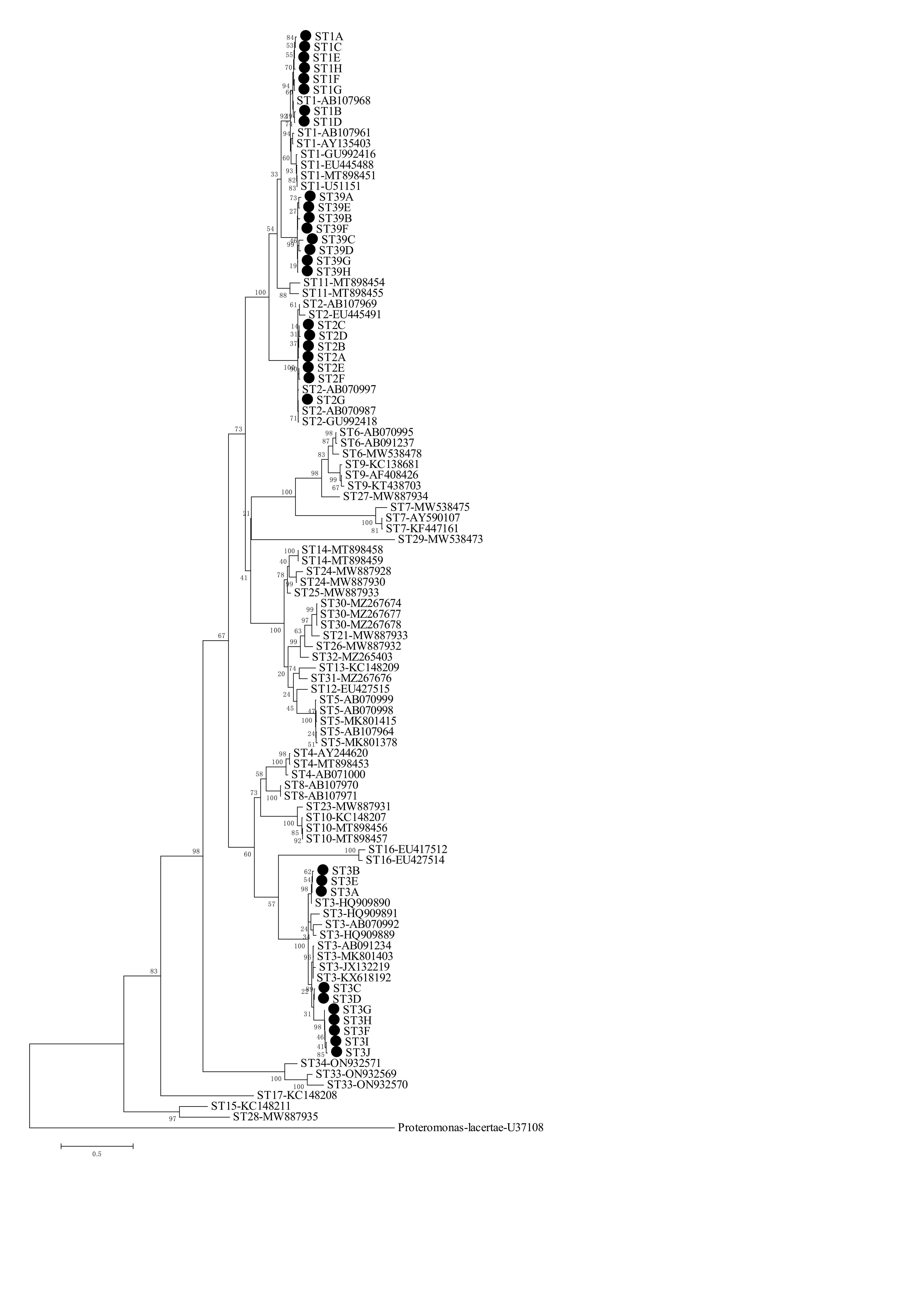

Supplement: Supplementary file 3 — Additional file 3: Figure S1. Phylogenetic relationships among Blastocystis SSU rRNA gene sequences (1690 bp) in the present study (represented with a black circle) and representative reference sequences. Proteromonas lacertae was used as outgroup taxon to root the tree. Analysis was conducted by a maximum likelihood method. Genetic distances were calculated using the Kimura two-parameter model. [file 13071_2023_5691_MOESM3_ESM.jpg]
